# Analysis of the cytotoxicity and bioactivity of CeraSeal, BioRoot™ and AH Plus^®^ sealers in pre-osteoblast lineage cells

**DOI:** 10.1186/s12903-024-04021-2

**Published:** 2024-02-22

**Authors:** Luciano Aparecido de Almeida-Junior, Giuliana de Campos Chaves Lamarque, Henry Herrera, Maya Fernanda Manfrin Arnez, Francine Lorencetti-Silva, Raquel Assed Bezerra Silva, Léa Assed Bezerra Silva, Francisco Wanderley Garcia Paula-Silva

**Affiliations:** 1 Present Address: Department of Clinics and Surgery, School of Dentistry, Federal University of Alfenas, Alfenas, Minas Gerais Brazil; 2 Department of Pediatric Dentistry, Ribeirão Preto School of Dentistry, University of São Paulo, Av. do Cafe s/n, Monte Alegre, Ribeirão Preto, São Paulo CEP: 14040-904 Brazil; 3 Universidad Católica de El Salvador, San Salvador, El Salvador; 4 School of Denstistry, Universidade de Rio Verde, Rio Verde, Goiás, Brazil

**Keywords:** Root canal filling materials, Cytotoxicity, Inflammation, Biomineralization, Osteoblasts

## Abstract

**Background:**

The objective of the present study was to evaluate in vitro the cytotoxicity and bioactivity of various endodontic sealers (CeraSeal, BioRoot™ and AH Plus^®^) in pre-osteoblast mouse cells (MC3T3 cells).

**Methods:**

MC3T3 cells (ATCC CRL-2594) were plated in 1 × 10^4^ cells/well in 96-well plates in contact with endodontic sealers at concentrations of 1:10 and 1:100. Cell viability was evaluated by MTT assay after 24 and 48 h. In addition, sealer bioactivity was measured by RT-PCR for mediator of inflammation (*Tnf, Ptgs2*) and mineralization (*Runx2, Msx1, Ssp1 and Dmp1*) after 24 h and by Alizarin Red S Assay of mineralization after 28 days. Data were analyzed using one-way ANOVA followed by the Tukey’s post-test at a significance level of 5%.

**Results:**

BioRoot™ presented 24-hour cytotoxicity (*p* < 0.05) at 1:10 concentration. In the period of 48 h, no endodontic cement was cytotoxic to the cells compared to the control (*p* > 0.05). TNF-α gene expression was induced by AH Plus^®^ (*p* < 0.05), while *Ptgs2* was induced by the CeraSeal and BioRoot™ (*p* < 0.05). The expression of *Runx2* was stimulated by BioRoot™ and AH Plus^®^ (*p* < 0.05). In contrast, the expression of *Dmp-1* *Dmp1* was higher for the CeraSeal and BioRoot™ (*p* < 0.05). Nonetheless, the sealers did not impact the formation of mineralization nodules (*p* > 0.05).

**Conclusion:**

CeraSeal, BioRoot™ and AH Plus^®^ sealers were not cytotoxic to MC3T3 cells within 48 h, but differentially induced the expression of genes related to inflammation and mineralization without impacting biomineralization by the cells.

## Background

Dental pulp contains important structures for maintaining the vitality of the tooth, such as blood vessels, cells, collagen fibers and nerves [1–6]. When this tissue loses its defense capacity after injuries (chemical, physical, mechanical, thermal or microbial), pulp necrosis occurs and a possible therapy for that is non-surgical root canal treatment [6]. Non-surgical root canal treatment involves the removal of necrotic tissue, cleaning and decontaminating of the root canal and placement of a compatible material with human tissues that seals and promotes repair, which is usually the gutta percha cone with an endodontic filling sealer [6, 7]. Endodontic sealers are materials that fill the space between the dentin walls and gutta-percha points along the entire root canal [8]. Their properties should include dimensional stability, biological sealing and biocompatibility with the adjacent tissues [9]. Sealers are classified according to their chemical constituents in zinc oxide and, eugenol, calcium hydroxide, glass ionomer, silicone, resin and bioceramic-based sealers [8].

These varieties of chemical constituents can alter the biological and physicochemical characteristics of filling cements, in addition to their bioactivity [9]. Filling cements, when bioactive, interact with cells in the apical and periapical region by direct contact or by diffusion to the tissues. Currently, one of the greatest challenges in endodontics is finding materials that promote the formation of mineralized tissue, i.e. that are bioactive [10–12].

AH Plus^®^ is an endodontic sealer based on epoxy resin, considered the gold standard for comparison with other filling sealers since it has excellent physical-chemical properties, strength and dimensional stability [13]. It is currently one of the most studied materials both in in vitro and in vivo [14–16]. However, its bioactivity within tissues is still limited [17, 18]. With this issue in mind, bioceramic sealers were developed to improve the response of the apical and periapical tissue [18]. This compound may contain alumina, zirconia, bioactive glass, glass-ceramics, hydroxyapatite and calcium phosphates and are grouped in cements based on calcium silicate or calcium phosphate [8].

BioRoot™ is a bioceramic material developed as a calcium silicate-based root canal sealer with high solubility and without cytotoxic effects in vitro and mild antibacterial activity [19]. It can be used in the single-cone obturation technique or by lateral condensation [20]. BioRoot™ induces the synthesis of angiogenic and osteogenic growth factors by periodontal ligament cells, has the ability to stimulate mineralized tissue formation and presents antimicrobial action, but demonstrated significantly more solubility than other sealers [21–25].

CeraSeal (Meta Biomed Co., Cheongju, Korea), is a pre-mixed calcium silicate-based bioceramic material. Because it was more recently released, knowledge in the literature about this material is scarce in both in vitro and in vivo studies [12, 26]. Calcium silicate-based cements favor the biomineralization process [27]. Studies show that bioceramic materials are more biocompatible and have a greater capacity to induce osteoblast differentiation compared to AH Plus [8, 18].

It is extremely important to evaluate the cytotoxicity, bioactivity and reparative and/or regenerative potential of an endodontic filling material. Although the cytotoxicity of calcium silicate-based materials has been studied in periodontal ligament cells [12], knowledge of the CeraSeal material is limited in the literature regarding the inflammatory and mineralizing process in osteoblastic cells. Therefore, the aim of the present study was to evaluate in vitro the cytotoxicity and bioactivity of CeraSeal, BioRoot™ and AH Plus^®^ endodontic sealers in pre-osteoblast mouse cells.

## Methods

### Sample preparation

The present study was carried out in accordance with ISO 10993-5: 2009 [28]. The extraction method was used to place the materials Cera Seal (calcium silicate; CSL 1,908,061), BioRoot™ RCS (tricalcium silicate; B23378-181011) and AH Plus^®^ (Epoxy Resin; 291,293 J) materials in contact with cells tested. As previously described [29], prefabricated matrices with 2 mm in diameter and 3 mm in height were used. The detailed composition of the materials is described in Table 1. The manipulation of the materials was carried out according to the manufacturer’s instructions on a sterile glass slab in a laminar flow cabinet and introduced into the sterile matrices using sterile instruments for a volume of 37.68 mm^3^.

**Table 1:** Description of the materials used (Name, composition and manufacturer)

Material	Composition	Manufacturer
CeraSeal	Calcium silicates, zirconium oxide, thickening agent.	MetaBiomed, Korea
Bio Root RCS	Powder based on tricalcium silicate, zirconium oxide and povidone. Aqueous solution of calcium chloride and polycarboxylate.	Septodont, France
AH Plus sealer	Paste A- Bisphenol-A Epoxy Resin, Bisphenol-F Epoxy Resin Calcium Tungstate Zirconium Oxide Silica, Iron Oxide Pigments. Paste B- Dibenzyldiamine Aminoadamantane, Tricyclodecane Diamine, Calcium Tugstate, Zirconium Oxide, Silica, Silicone Oil.	Dentsply Maillefer, Switzerland

The prepared materials were kept in the laminar flow cabinet under ultraviolet light (UV) for 1 h to eliminate contamination and then placed separately in sterile plastic tubes. Next, they were placed in polypropylene tubes with 2 ml of Alpha Modified Minimal Essential Medium (α MEM- Gibco, Invitrogen, Grand Island, NY, USA) and stored at 37 °C for 24 h before the experiment. The extract was filtered using a 0.22 μm Millipore filter, the remaining materials were discarded, and 1:10 and 1:100 serial dilutions were prepared from the initial extracts (1:1) [29]. The 1:1, 1:10, and 1:100 solutions were kept in refrigerated storage until use.

### Cell culture

Mouse pre-osteoblast cell line (MC3T3 – ATCC CRL-2594 – Banco de Células do Rio de Janeiro - BCRJ) were maintained in α-MEM, supplemented with 5% fetal bovine serum (FBS) (Fetal Bovine Serum, Certified, Heat-Inactivated, Gibco, Invitrogen), penicillin and 1% streptomycin (Penicillin-Streptomycin, Gibco, Invitrogen). The cells were kept in a humidified incubator at 37 °C with 5% CO_2_. The medium was changed every 2 days. Cells were used between the 5th and 10th passage.

For the experiments, 1 × 10^4^ cells/well were plated into 96-well cell culture plates (Cell Wells; Corning Glass Workers, NY, USA), and the cells were left to attach overnight in an incubator. The experiment was carried out in triplicate.

### Cell viability - MTT colorimetric assay

Cell viability was evaluated using a MTT assay according to the manufacturer’s instructions. The cells were stimulated for 24 and 48 h according to the division of the groups (CeraSeal 1:10/CeraSeal 1:100/BioRoot™ RCS 1:10/BioRoot™ RCS 1 :100/AH Plus^®^ sealer 1:10/AH Plus^®^ sealer 1:100/Positive control - DMSO/Negative control - cells cultured without FBS). The supernatants were discarded and 10 µL of MTT (3-(4.5-dymethylthiazol-2-yl)-2.5-diphenyltetrazoluim bromide, Sigma-Aldrich Co., Catalog number M2128) supplemented with 150 µL Roswell Park Memorial Institute medium (RPMI, catalog number 11.835.030, 500 ml). The MTT solution was dissolved in colorless RPMI (0.5 mg/mL), filtered and sterilized using a 0.22 μm Millipore filter. The solution was added to each well) and incubated for 4 h at 37ºC. Next, the MTT solution was removed, and dimethyl sulfoxide (DMSO – Fisher Scientific, Hampton, VA, USA) was added and kept for 30 min at room temperature to completely dissolve the formazan crystals. The absorbance reading was determined in a spectrophotometer device with a wavelength set at 570 nm (mQuanti; Bio-tek Instruments, Inc, Winooski, VT).

### Reverse transcription polymerase chain reaction - RT-PCR

The sealers dilution of 1:100 was selected for assay by qRT-PCR, after cell viability evaluation. Thus, mRNA levels for *Tnf, Ptgs2, Runx2, Msx1, Ssp1* and *Dmp1* were evaluated. As a result, after stimulation with extracts of the sealers, the cells were harvested and total RNA was extracted using the RNeasy® Mini kit (Qiagen Inc., Valencia, USA) according to the manufacturer’s instructions and quantified using a NanoDrop 2000 spectrophotometer (Thermo Fisher Scientific Inc., Wilmington, MA, USA). Next, cDNA synthesis was performed in a thermal cycler (Veriti® Thermal Cycler, Applied Biosystems) by the polymerase chain reaction. Primers and probes for *Tnf* (Mm 00443258_m1), *Ptgs2* (Mm00478374_m1), *Runx2* (Mm00501584_m1), *Msx1* (Mm00440330_m1), *Ssp1* (Mm00436767_m1), and *Dmp1* (Mm01208363_m1) were used. Quantitative reverse transcriptase-polymerase chain reactions (qRT-PCR) were done in duplicate using the TaqMan® system in a StepOne Plus® real-time PCR system (StepOne Plus® Real-Time PCR System, Applied Biosystems) using the following cycling program: 95 °C for 20 s, followed by 40 cycles at 95 °C for 1 s and 60 °C for 20 s. All protocols were performed according to the manufacturers’ instructions. Primer-probe pairs were obtained commercially, and thus their sequences are not available. (TaqMan Gene Expression Assay; Applied Biosystems, Foster City, CA). The quantifications were normalized using glyceraldehyde-3-phosphate dehydrogenase *(Gapdh)* and beta-actin *(Actb)* as reference genes. For each gene, relative expression was calculated by the 2 ^−ΔΔCt^ method.

### Mineralization assay

Cells were cultured for 28 days under mineralization conditions in α-MEM supplemented with 10 mM β-glycerophosphate (Sigma), 50 µg/mL ascorbic acid (Sigma), 5% FBS, penicillin and 1% streptomycin (Penicillin-Streptomycin, Gibco, Invitrogen). For the experiments, 2 × 10^4^ cells/well were plated into 96-well cell culture plates. After subconfluence, cultures were stimulated with CeraSeal 1:100, BioRoot™ RCS 1:100 and AH Plus^®^ 1:100 filling materials. The medium was changed every 3 days and the cell culture progression was assessed by brightfield microscopy. Alizarin Red S solution was added following the previously described protocols [30]. Briefly, cultures were fixed with 70% ethanol for 10 min and stained with 2% alizarin red solution (pH 4,0) for 5 min at room temperature. To quantify the degree of calcium accumulation in the mineralized extracellular matrix, alizarin red–stained cultures were incubated with 100 mM cetylpyridinium chloride (Sigma) for 1 h to release the calcium-bound dye into the solution under agitation. The absorbance of the released dye was measured at 570 nm using a spectrophotometer and normalized by the total protein concentration in the culture.

### Statistical analysis

Statistical analysis was performed using the GraphPad Prism 8.0 Software (Prism, Chicago, IL, USA), using one-way ANOVA followed by the Tukey’s post-test, adopting a significance level of 5%.

## Results

### AH Plus^®^, Bio Root™ and CeraSeal impacted cell viability 

**Fig. 1:**
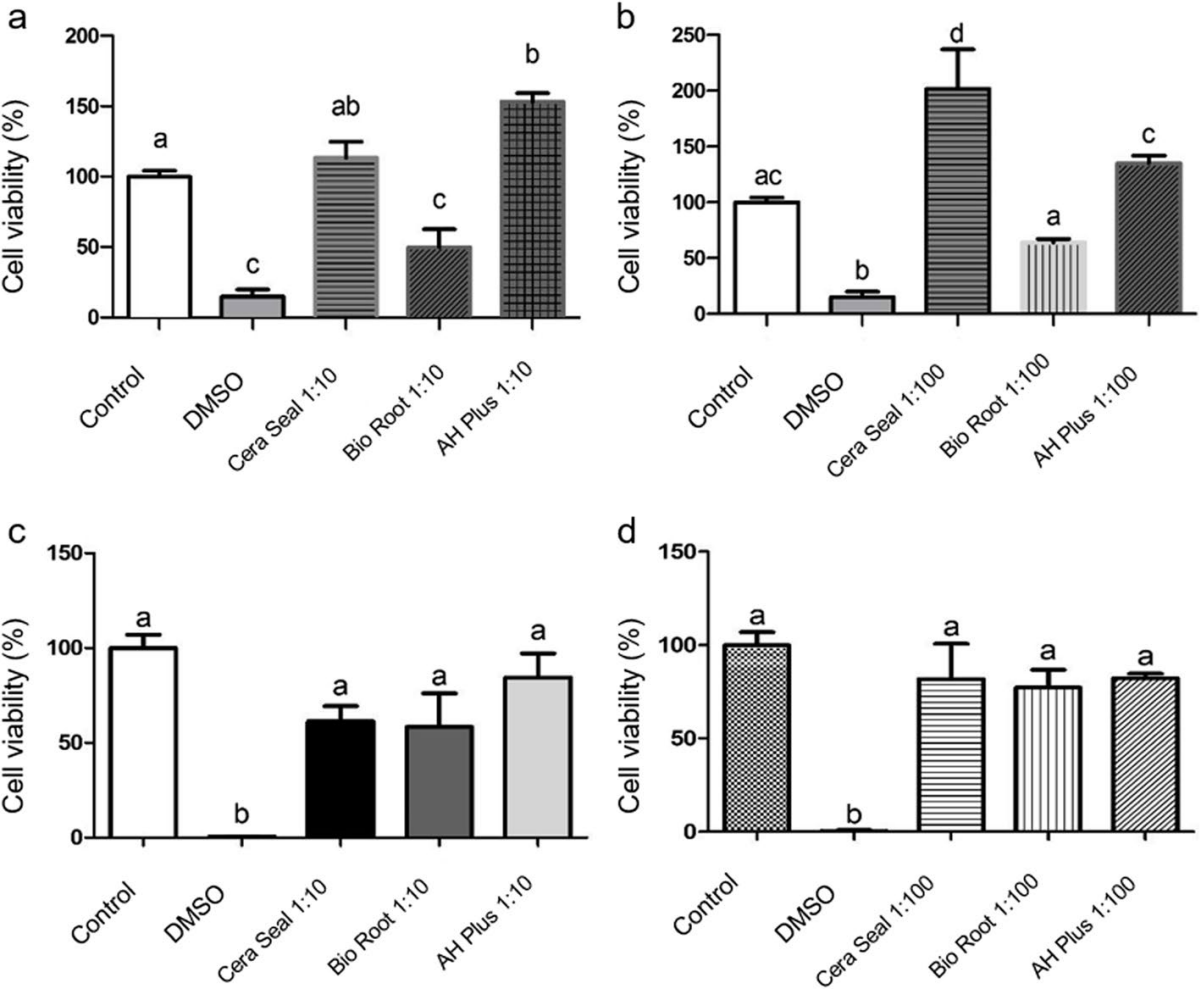
Percentage of cell viability by the MTT assay in a 24-hour period - control, DMSO, CeraSeal, Bio Root™ and AH Plus® groups at a concentration of 1:10 with αMEM (**a**). Percentage of cell viability by the MTT assay in a 24-hour period - control, DMSO, CeraSeal, Bio Root™﻿ and AH Plus® groups at a concentration of 1:100 with αMEM (**b**). Percentage of cell viability by the MTT assay in a 48-hour period - control, DMSO, CeraSeal, Bio Root™﻿ and AH Plus® groups at a concentration of 1:10 with αMEM (**c**). Percentage of cell viability by the MTT assay within 48 h - control, DMSO, CeraSeal, Bio Root™ and AH Plus® groups at a concentration of 1:100 with αMEM (**d**). Note: Different lowercase letters indicate that there is a statistical difference between the groups

Cell viability analysis using the MTT method after 24 h showed a reduction in contact with Bio Root™ cement (*p* < 0.05) at 1:10 concentration (*p* < 0.05), while the CeralSeal and AH Plus^®^ sealers were similar (*p* > 0.05) or higher (*p* < 0.05) than the control, respectively (Fig. 1a and b). After 48 h, the cell viability was similar in Bio Root™ and AH Plus^®^ groups (*p* > 0.05), except in the group stimulated with Cera Seal or DMSO (positive control; *p* < 0.05) (Fig. 1c and d).

### Root canal filling materials stimulate the expression of genes related to inflammation and mineralization

**Fig. 2:**
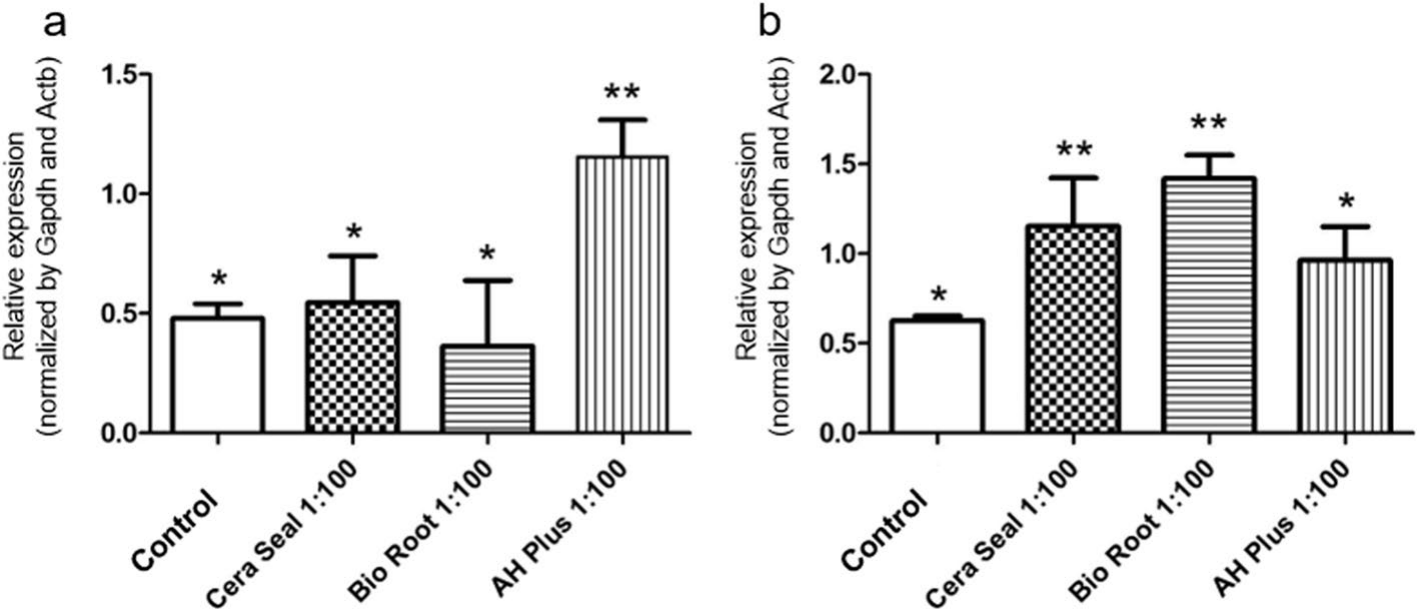
mRNA expression for *Tnf* (**a**) and *Ptgs2* (**b**) within 24 h after stimulation with CeraSeal, Bio Root™ and AH Plus^®^ materials at a concentration of 1:100 or control (cells maintained with conventional culture medium). Note: Different symbols indicate that there is a statistical difference between the groups

AH Plus^®^ sealer up-regulated the expression of *Tnf* (*p* < 0.05). On the other hand, CeraSeal and BioRoot™ stimulated-cells had gene expressions similar to the control (*p* > 0.05) (Fig. 2a). CeraSeal and BioRoot™ sealers up-regulated *Ptgs2* expression compared to the control and AH Plus^®^ (*p* < 0.05) (Fig. 2b).

**Fig. 3:**
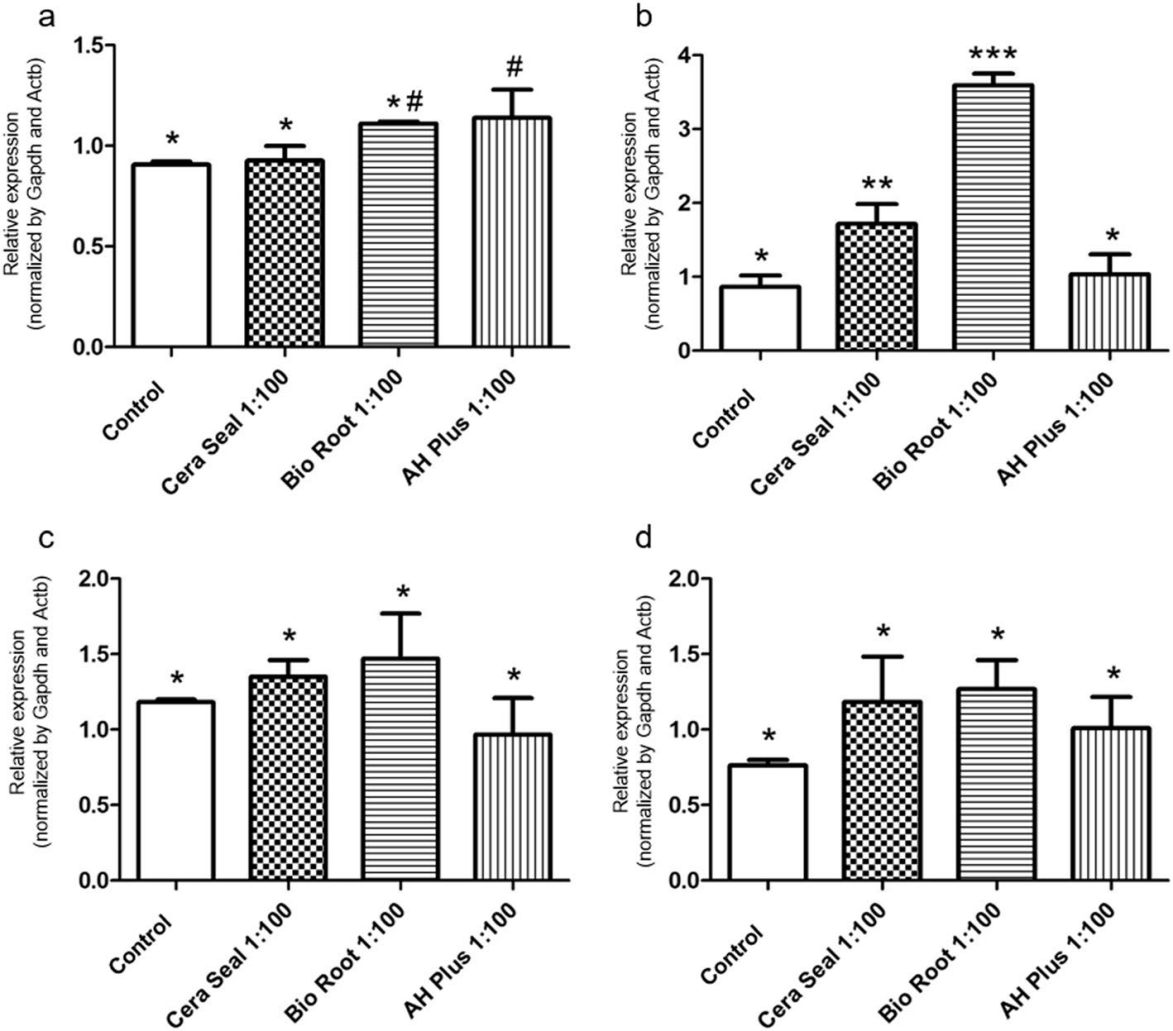
mRNA expression for *Runx2* (**a**), *Dmp1* (**b**), *Msx1* (**c**) and *Spp1* (**d**) within 24 h after stimulation with CeraSeal, Bio Root™ and AH Plus® materials at a concentration of 1:100 or control (cells maintained with conventional culture medium). Note: Different symbols indicate that there is a statistical difference between the groups

AH Plus^®^ sealer induced *Runx2* expression (*p* < 0.05), while CeraSeal and BioRoot™ stimulated-cells presented gene expression similar to the control (*p* > 0.05) (Fig. 3a). Dentin matrix protein (*Dmp1*) synthesis was stimulated by CeraSeal and BioRoot™ differently from the AH Plus^®^ or the control group (*p* < 0.0001) (Fig. 3b). The expression of *Msx1* and *Spp1* were not modulated by any sealer (*p* = 0.1769) (Fig. 3c and d).

### Filling materials did not impact the formation of mineralization nodules

**Fig. 4:**
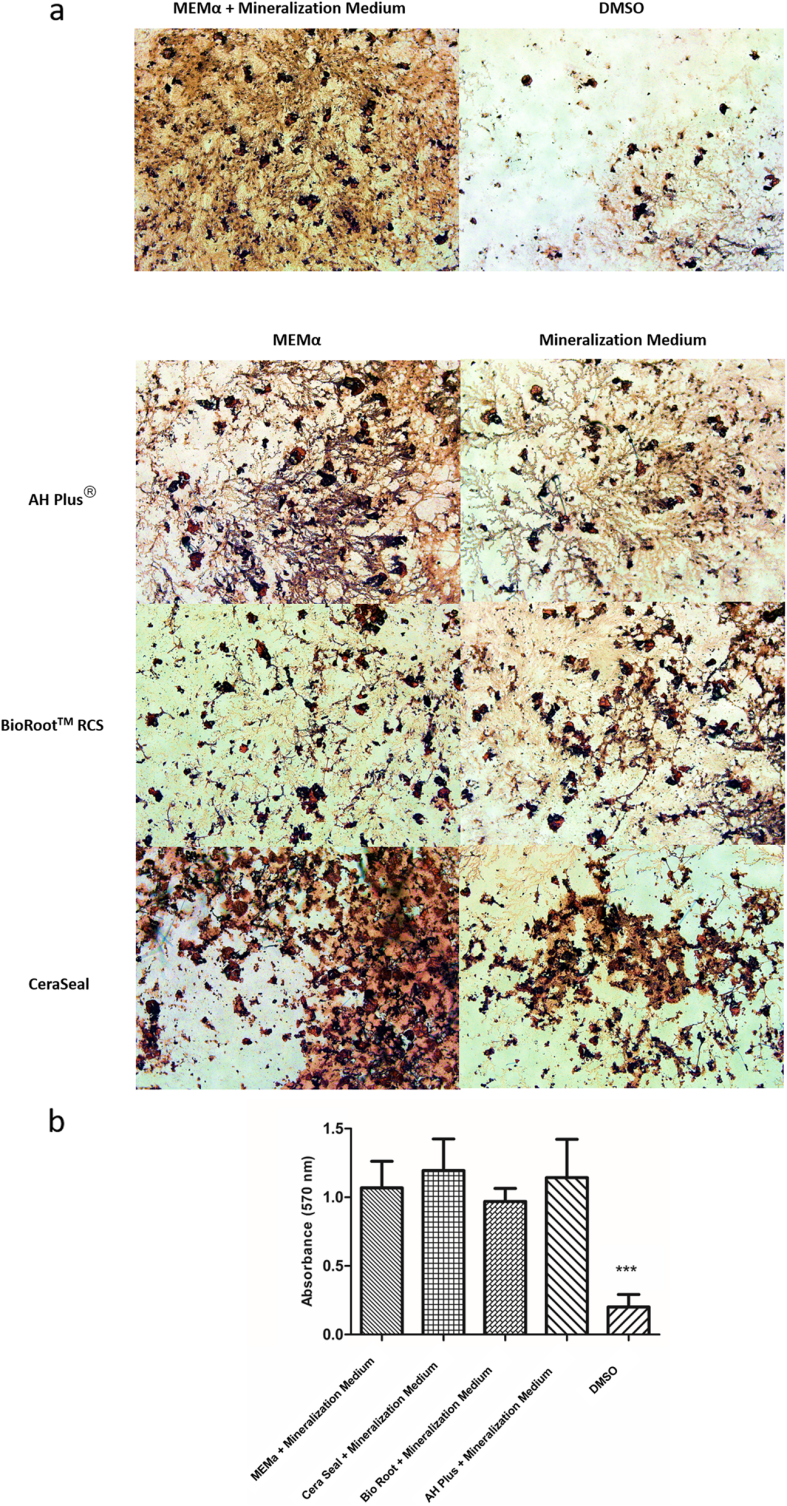
Representative photomicrographs of Alizarin Red S Assay. Original 20x magnification (scale = 50 μm) (**a**). Formation of mineralization nodules at 28 days after stimulation with the CeraSeal, Bio Root™ and AH Plus® materials at a concentration of 1:100 or control (cells maintained with culture medium containing beta glycerophosphate and ascorbic acid) and DMSO (**b**). Note: Different symbols indicate that there is a statistical difference between the groups. Mineralization medium= beta-glycerophosphate and ascorbic acid

After 28 days, the mineralization nodules formation was similar among all groups compared to the control (*p* > 0.05), except for the DMSO, which showed a reduced formation of mineralization nodules (*p* < 0.0001) (Fig. 4).

## Discussion

In general, the evaluated sealers were not cytotoxic to the MC3T3 cells and did not induce the formation of mineralization nodules, but induced the gene expression related to inflammation and mineralization. Previous studies used different cell types such as fibroblasts, osteoblasts and periodontal ligament cells for evaluation of those materials [12, 31, 32]. However, we intended to analyze in vitro the cytotoxicity and repair potential of filling materials that are in contact with cells in the apical and periapical regions. Cells from osteoblast lineages were developed as models for in vitro investigation for studies of cell differentiation, cytokine and hormone production, protein synthesis and secretion, understanding of molecular mechanisms of diseases and drug pharmacokinetics [33].

MC3T3-E1 is a well-known osteoblast lineage that represents a pre-osteoblast phenotype [33]. MC3T3 cells have high proliferative capacity and mineralization potential when stimulated with growth factors, ascorbic acid or mineralization enzymes, in addition, at high passages they become senescent similar to human tissues. These factors make these cells attractive for in vitro studies on topics related to the repair and/or regeneration process of mineralized tissue [33, 34].

​The cytotoxicity of the endodontic sealers can change with time and depends on the material concentration [35, 36]. This may explain the difference in cell viability in 24 and 48 h and also in 1:10 and 1:100 concentrations observed in the present study. Our results demonstrate that only the BioRoot™ showed a reduction in cell viability at 24h and that after 48 h, it did not show differences compared to the other groups. Previously it was demonstrated that sealers based on calcium silicate, epoxy resin and zinc oxide and eugenol were cytotoxic in 24 h, but after 1 week, calcium-silicate based sealers and AH Plus did not demonstrate cytotoxicity [37]. The root canal bioactive sealers have demonstrated the ability to minimize acute inflammatory responses and promote quicker periapical healing [38]. However, in fibroblast cells, CeraSeal showed a reduction in cytotoxicity from 24 h to 7 days, unlike AH Plus, which demonstrated to be cytotoxic in all periods evaluated [39].

Resin-based endodontic sealers may be more cytotoxic to cells such as fibroblasts [40], human dental pulp stem cells [41], and periodontal ligament cells [42–44]. The present study is the first to demonstrate that AH Plus^®^ is not cytotoxic to MC3T3 osteoblast lineage cells. Interestingly, there is evidence that epoxy resin-based endodontic sealers, such as AH APlus, have compatibility with alveolar osteoblasts [45]. However, AH Plus^®^ induced higher Tnf-α gene expression compared to the BioRoot™ RCS and CeraSeal sealers, which may have negative effects of the inflammatory process. Some studies demonstrated that high levels of Tnf-α may enhance osteoclast formation and bone resorption and induce apoptosis of osteoblast cells [46–48]. The calcium hydroxide–based Sealapex Xpress sealer inhibited the expression of TNF-α, while the methacrylate-based resin sealer (Real Seal XT) caused the induction of TNF-α within 24 h [49].

Previous studies have shown that calcium silicate-based sealers induce positive responses, such as precipitation of hydroxyapatite, influence in cellular plasticity, differentiation of stem cells into osteoblasts, odontoblasts and cementoblasts and effectively act in the apical repair process [50–52]. The present results demonstrate that these materials have in vitro ability to induce genes related to bone mineralization. Previously, it was demonstrated that Ceraseal showed greater calcium release and alkalizing activity when compared to the NeoSealer Flo [53].

Bioceramic materials that are calcium silicate-based promote interactions with bone and dental tissue cells, however, their effects on different cell types are different [54]. We used some osteo/odontogenic markers in the presence of epoxy-resin and bioceramics endodontic materials, such as *Runx2*, an early-stage osteogenesis marker, *Ssp1* and *Dmp1* markers associated with the formation and mineralization by odontoblasts [54]. The present study showed that gene expression for these markers in the BioRoot™ group showed the highest expression for *Dmp1*, followed by the CeraSeal. AH Plus^®^ and BioRoot™ RCS induced higher expression of *Runx2* and there was no difference for *Ssp1* expression in osteoblast cells.

We observed that endodontic filling sealers do not interfere in the mineralization process in a negative way, corroborating with a previous observation [37]. Evaluating the in vitro response of cytotoxicity and biocompatibility is essential in order to clarify which future processes the filling sealers will interfere in a beneficial or harmful way in the cells. The present study evaluated endodontic cements based on epoxy resin and bioceramics based on calcium silicate in osteoblast cells and demonstrated the potential for differentiation of cells in contact with these materials. Since it is an in vitro study, it has some limitations, the main one being extrapolation to clinical practice. However, it is of fundamental importance to have knowledge regarding the physicochemical aspects of these materials and how that might affect inflammation and mineralization processes so that new studies become necessary to elucidate the molecular mechanisms in the formation of mineralized tissue.

## Conclusion

AH Plus^®^, CeraSeal and BioRoot™ RCS sealers were not cytotoxic to MC3T3 cells, but differentially induced the expression of genes related to inflammation and mineralization without impacting the biomineralization function of these cells.

## Data Availability

No datasets were generated or analysed during the current study.

## Abbreviations

Tnf-α: Tumor necrosis factor-alpha
*Ptgs2*: Prostaglandin-endoperoxide synthase 2
*Runx2*: Runt-related transcription factor 2
*Ssp1*: Osteopontin
*Dmp1*: Dentin matrix protein 1
ISO: International Organization for Standardization
UV: Ultraviolet light
α MEM: Alpha Modified Minimal Essential Medium
FBS: Fetal bovine serum
CO_2_: Carbon dioxide
DMSO: Dimethyl sulfoxide
MTT: 3-(4,5-dymethylthiazol-2-yl)-2,5-diphenyltetrazoluim bromide
*RT-PCR*: Reverse transcription polymerase chain reaction
*Gapdh*: Glyceraldehyde 3-phosphate dehydrogenase
*Actb*: Beta actin
